# Comprehensive multi-modality assessment of regional and global arterial structure and function in adults born preterm

**DOI:** 10.1038/hr.2015.102

**Published:** 2015-09-24

**Authors:** Henry Boardman, Katherine Birse, Esther F Davis, Polly Whitworth, Veena Aggarwal, Adam J Lewandowski, Paul Leeson

**Affiliations:** 1 Radcliffe Department of Medicine, Division of Cardiovascular Medicine, University of Oxford, Oxford, UK

**Keywords:** arterial stiffness, blood pressure, preterm birth

## Abstract

Preterm birth is associated with higher blood pressure, which could be because preterm birth alters early aortic elastin and collagen development to cause increased arterial stiffness. We measured central and conduit artery size and multiple indices of arterial stiffness to define the extent and severity of macrovascular changes in individuals born preterm. A total of 102 young adults born preterm and 102 controls who were born after an uncomplicated pregnancy underwent cardiovascular magnetic resonance on a Siemens 1.5 T scanner to measure the aortic cross-sectional area in multiple locations. Ultrasound imaging with a Philips CX50 and linear array probe was used to measure carotid and brachial artery diameters. Carotid-femoral pulse wave velocity and the augmentation index were measured by SphygmoCor, brachial-femoral pulse wave velocity by Vicorder and aortic pulse wave velocity by cardiovascular magnetic resonance. The cardio-ankle vascular index (CAVI) was used as a measurement of global stiffness, and ultrasound was used to assess peripheral vessel distensibility. Adults born preterm had 20% smaller thoracic and abdominal aortic lumens (2.19±0.44 *vs.* 2.69±0.60 cm^2^, *P*<0.001; 1.25±0.36 *vs.* 1.94±0.45 cm^2^, *P*<0.001, respectively) but similar carotid and brachial diameters to adults born at term. Pulse wave velocity was increased (5.82±0.80 *vs.* 5.47±0.59 m s^−1^, *P*<0.01, 9.06±1.25 *vs.* 8.33±1.28 m s^−1^, *P*=0.01, 5.23±1.19 *vs.* 4.75±0.91 m s^−1^, *P*<0.01) and carotid distensibility was decreased (4.75±1.31 *vs.* 5.60±1.48 mm Hg^−1^10^3^, *P*<0.001) in this group compared with the group born at term. However, the global and peripheral arterial stiffness measured by CAVI and brachial ultrasound did not differ (5.95±0.72 *vs.* 5.98±0.60, *P*=0.80 and 1.07±0.48 *vs.* 1.19±0.54 mm Hg^−1^10^3^, *P*=0.12, respectively). Adults who are born preterm have significant differences in their aortic structure from adults born at term, but they have relatively small differences in central arterial stiffness that may be partially explained by blood pressure variations.

## Introduction

Advances in neonatal care have led to a rise in perinatal survival of those born preterm.^1, 2^ Interestingly, follow-up of these individuals into early adulthood has demonstrated that they tend to have higher blood pressure as they age, compared with those born at term, with the increase proportional to their degree of prematurity.^3, 4, 5^ This trend may be due to the premature transition of their cardiovascular system to an *ex utero* physiological state,^6, 7^ which leads to adverse early cardiovascular growth and development. For example, in experimental models, the elastin and collagen maturation that occurs in the arterial wall during late gestation is prematurely halted by an early transition to postnatal physiology.^8^ Perinatal interventions used in preterm neonates, such as antenatal glucocorticoids and intravenous lipids, are also known to modify connective tissue architecture and function in the aortic arch and abdominal aorta, respectively.^9, 10^ Therefore, permanent changes in arterial size and stiffness may underlie the observed increase in hypertension risk in the offspring of women with complicated pregnancies,^11^ particularly in individuals who are born preterm.^12, 13^

Apparently consistent with this hypothesis, several studies have reported reduced vessel size^4, 14, 15, 16^ and increased arterial stiffness^16, 17, 18^ in preterm offspring. However, others have failed to replicate these findings.^3, 19, 20, 21^ This variability may have occurred because several studies had small sample sizes or because elastin content and exposure to perinatal hemodynamic stress varied between arterial locations, resulting in localized changes in arterial stiffness. Additionally, pulse wave velocity is known to change with blood pressure at the time of measurement, and stiffness has not been assessed with more recent indices known to be independent of blood pressure. Therefore, we compared central and conduit artery size in a large group of adults born preterm to measurements in term-born adults. Then, we characterized their global arterial stiffness using pulse wave velocity and the cardio-ankle vascular index (CAVI) to take into account the physiological impact of the known variation in blood pressure.

## Methods

### Study population

A cohort of individuals born preterm between 1982 and 1985 has been prospectively followed since recruitment at birth to a randomized feeding intervention trial.^6, 7, 9, 10^ At follow-up during adolescence, 240 of the initial 926 participants (birthweight <1850 g) agreed to be contacted about future studies. We performed detailed cardiovascular phenotyping on 102 of the participants at age 23–28 years.^6, 7, 9, 10^ For this study, 102 adults who had been born at term to women with uncomplicated pregnancies, with frequencies matched to the age and sex distributions of the preterm adults, were recruited to undergo identical investigations. The study was registered with ClinicalTrials.gov (NCT01487824), and the protocols were approved by the relevant ethics committee (Oxfordshire Research Ethics Committee A: 06/Q1604/118). All participants provided signed informed consent.

### Perinatal data and cardiovascular risk factors

Details of sample and data collection have been previously reported.^6, 7, 9, 10, 22^ Briefly, preterm perinatal data were collected from medical records during the first weeks of life, with participant maternal recall used to validate birth history in term-born adults.^6, 7, 9, 10^ Blood pressure was measured after 10 min of supine rest based on an average of the last two out of three left brachial blood pressure recordings using an automatic digital monitor (HEM-705CP, OMRON, Japan).^6, 7, 9, 10^ Anthropometry measurements included height, weight and skinfold thickness. Medical and lifestyle information was collected using a questionnaire.^23, 24^ Blood samples were collected following a 12 h overnight fast and were centrifuged and separated within 30 min for storage at −80 °C before analysis. Fasting lipid profiles and metabolic measurements were conducted at the John Radcliffe Hospital (Oxford, UK) Biochemistry Laboratory using validated clinical assays.

### Arterial structure

#### Cardiovascular magnetic resonance

Cardiovascular magnetic resonance was performed on a 1.5 T (Siemens Sonata, Munich, Germany) scanner. The aortic cross-sectional area was measured from axial ECG-gated, steady-state free precession cine images acquired during breath holding at the level of the pulmonary artery (proximal descending aorta) and at the level of the second lumbar vertebra (abdominal aorta).^6, 7, 10^ Minimum aortic cross-sectional areas at the end-diastole were determined by using semi-automated edge detection algorithms (Matlab, Mathworks, Natick, MA, USA).^25^

#### Ultrasound imaging

Conduit vessel size was measured using a Philips CX50 ultrasound machine with a 12 MHz linear array transducer. The participant lay supine in a temperature-controlled room. Following 10 min of rest, ECG-gated longitudinal images of the right brachial artery were acquired 5–10 cm above the antecubital fossa. The carotid arteries were imaged with the patient lying flat and the head rotated to the opposite side from the carotid measure. The carotid bifurcation was identified, and then a longitudinal image was acquired that included the bulb and common carotid artery so that diameter could be measured 1 cm proximal to the bifurcation. The minimum arterial diameter at the end-diastole was measured offline using automated image analysis software (Vascular Analyzer; MIA, Coralville, IA, USA).

### Arterial stiffness

#### Cardiovascular magnetic resonance

The aortic pulse wave velocity was measured using free breathing and retrospectively ECG-gated with a spoiled gradient echo sequence. The velocity-encoding gradient for phase contrast was applied to measure the through-plane flow in the same position as the aortic size measurements at the level of the pulmonary artery and distal descending aorta. Image analysis was performed with Argus (Siemens Medical Solutions). Flow images were manually contoured as previously described.^26^ Aortic pulse wave velocity was calculated as the distance between the assessed sites (ascending aorta to distal descending aorta) measured on an aortic oblique sagittal image divided by the time delay between the arrival of the foot of the pulse wave at each site, which was determined as the intersection of the tangent of the upslope of the flow curve with the x-(time)axis.^26, 27, 28^

#### Non-Invasive measurements of arterial stiffness

The carotid-femoral pulse wave velocity was measured using applanation tonometry to obtain pressure waveforms of the carotid and femoral pulse (SphygmoCor; AtCor Medical, West Ryde, NSW, Australia). Brachial-femoral pulse wave velocity was measured using sphygmomanometer-derived indices (Vicorder, Skidmore Medical, Taunton, UK) with cuffs placed around the brachial and femoral arteries to identify the pulse arrival. With both techniques, the pulse wave velocity is derived from recordings of the time delay between the two measurement sites relative to the distance between them identified from predefined landmarks. The augmentation index was automatically measured from the pressure waveforms of the radial pulse obtained by applanation tonometry adjusted to a mean heart rate of 75 beats per minute (SphygmoCor; AtCor Medical). Minimum and maximum arterial diameters of the carotid and brachial arteries were measured offline from stored ultrasound image loops acquired over multiple cardiac cycles. Carotid and brachial diameters were measured using automated image analysis software (Vascular Analyzer). The distensibility of these arteries was quantified as the change in diameter relative to the minimum diameter proportional to the pulse pressure based on central and peripheral measurements recorded during the ultrasound image acquisition.^29^

#### Cardio-ankle vascular index (CAVI)

In a subgroup of participants, CAVI was measured with the VaSera VS-1500 (Fukuda Denshi, Tokyo, Japan). CAVI is an indicator of the stiffness of arteries from the origin of the aorta to the ankle of the lower leg. The measurement is developed from the stiffness parameter β and is independent of blood pressure at the time of measurement.^30, 31^

### Statistical analysis

Statistical analysis was performed using SPSS Version 20 (IBM, Armonk, NY, USA). Normality was assessed using visual assessment of histograms and the Shapiro–Wilk test. Comparisons between groups for continuous variables for normally distributed data were performed using a two-sided, independent-sample Student's *t*-test; the Mann–Whitney test was used for skewed data. The results are presented as the mean±s.d. *P*-values <0.05 were considered statistically significant. Comparisons between groups of categorical variables were performed using a *X*^2^-test. Linear regression was performed using backwards stepwise models.

## Results

### Study population

The demographic details of the study group and how it compares to the original cohort have been reported previously.^6, 7^ Briefly, at the time of the study visit, there were no significant differences between preterm and term-born individuals in the number of smokers, personal and family medical histories or lifestyle factors such as physical activity, diet or socioeconomic status. However, the adults born preterm were shorter, weighed more and had higher blood pressure than the young adults in the cohort born at term. Participants with CAVI-derived arterial measurements had characteristics similar to the full cohort (Table 1).

### Blood pressure

As previously reported for this cohort,^32^ brachial systolic and diastolic blood pressures were higher in adults born preterm than in adults born at term (systolic blood pressure, preterm-born adults: 121.3±10.9 mm Hg *vs.* term-born adults: 112.9±10.1 mm Hg, *P*<0.001 and diastolic blood pressure, preterm-born adults: 73.0±7.2 mm Hg *vs.* term-born adults 68.8±7.0 mm Hg, *P*<0.001, respectively). Similar differences were observed between the centrally measured systolic and diastolic blood pressures (systolic blood pressure, preterm-born adults: 108.0±10.0 mm Hg *vs.* term-born adults: 97.45±8.8 mm Hg, *P*<0.001 and diastolic blood pressure, preterm-born adults: 74.5±7.4 mm Hg *vs.* term-born adults 69.6±7.7 mm Hg, *P*<0.001, respectively). The amplification from central to brachial systolic blood pressure was 2 mm Hg lower in the preterm group than in the those born at term, but this difference did not reach statistical significance (13.6±6.8 mm Hg *vs.* 15.4±5.0 mm Hg, respectively, *P*=0.07). There was no evidence for significant amplification of diastolic blood pressure (−1.5±3.6  *vs.* −0.8±3.4 mm Hg, respectively, *P*=0.22)

### Arterial structure

**Figure 1:**
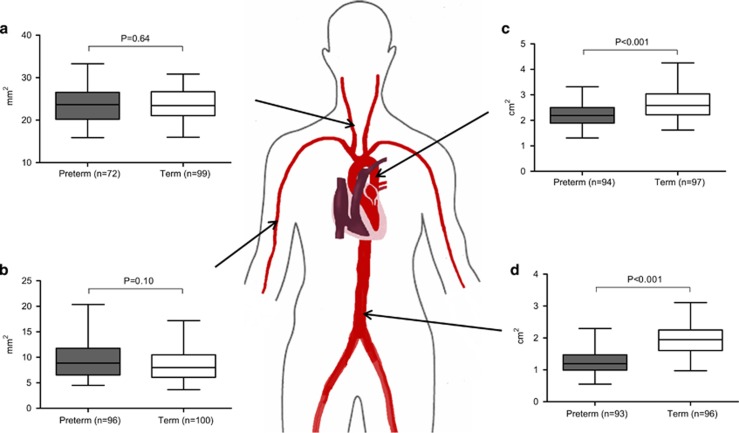
Arterial structure—preterm-born young adults (gray) demonstrated no statistically significant difference in carotid cross-sectional areas compared to term-born adults (white), (23.55±3.84 *vs.* 23.87±3.55 mm^2^, *P*=0.64) (**a**) or brachial cross-sectional areas compared to term-born adults (white), (9.34±3.25 *vs.* 8.57±2.91 mm^2^, *P*=0.10) (**b**). However, preterm-born young adults (gray) had reduced thoracic and abdominal aorta cross-sectional areas compared to term-born young adults (white), (2.19±0.44 *vs.* 2.69±0.60 cm^2^, *P*<0.001 (**c**) and 1.25±0.36 *vs.* 1.94±0.45 cm^2^, *P*<0.001) (**d**). A full color version of this figure is available at the *Hypertension Research* journal online.

There was no evidence of a reduced carotid artery lumen cross-sectional area in the participants born preterm (23.55±3.84 mm^2^ *vs.* 23.87±3.55 mm^2^, respectively, *P*=0.64, Figure 1a). The brachial artery lumen cross-sectional area was also not significantly different (9.34±3.25 mm^2^ *vs.* 8.57±2.91 mm^2^, respectively, Figure 1b) until after the adjustment for body size, which indicated a greater relative arterial cross-sectional area in those born preterm. However, young adults who were born preterm had significantly smaller lumen cross-sectional areas throughout the aorta, including the proximal thoracic aorta and abdominal aorta (2.19±0.44 *vs.* 2.69±0.60 cm^2^, *P*<0.001 and 1.25±0.36 *vs.* 1.94±0.45 cm^2^, *P*<0.001, respectively). These results were not substantially changed in a multivariate model that included measurements of body size and mean blood pressure.

### Arterial stiffness

**Figure 2:**
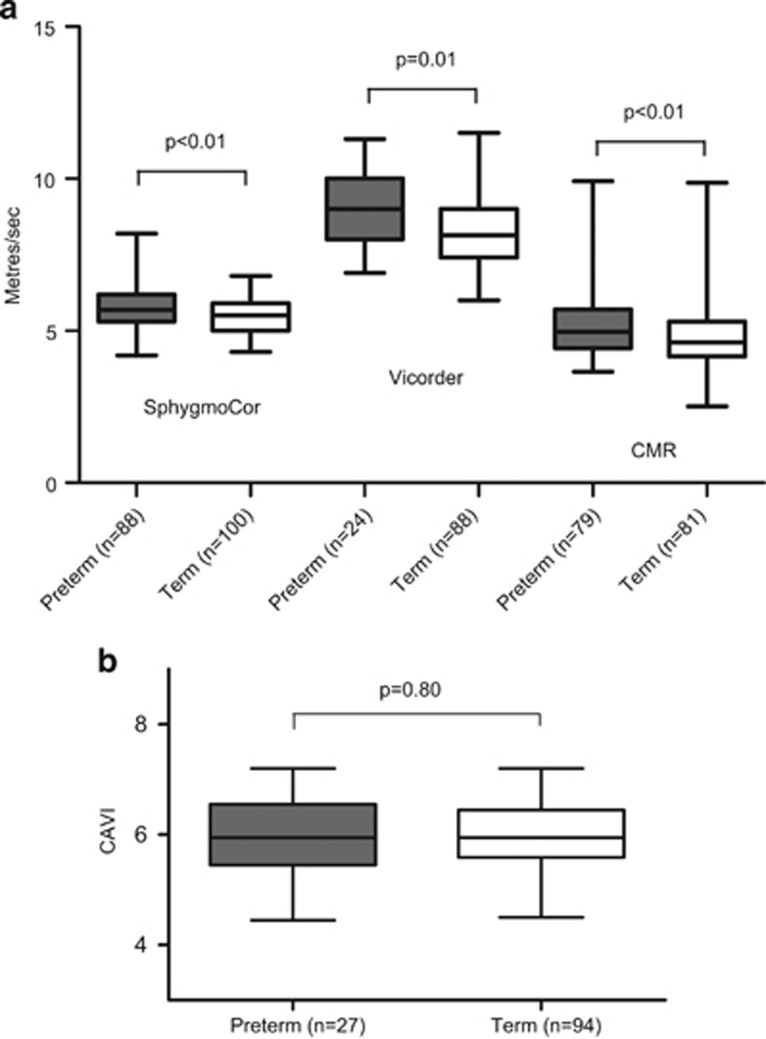
Arterial Stiffness—preterm-born young adults (gray) had increased measures of SphygmoCor, Vicorder and cardiovascular magnetic resonance pulse wave velocity compared with term-born young adults (white), (5.82±0.80 *vs.* 5.47±0.59 m s^−1^, *P*<0.01, 9.06±1.25 *vs.* 8.33±1.28 m s^−1^, *P*=0.01, and 5.23±1.19 *vs.* 4.75±0.91 m s^−1^, *P*<0.01) (**a**). Lack of differences in arterial stiffness was also demonstrated in preterm-born young adults (gray) by CAVI compared with term-born adults (white), (5.95±0.72 *vs.* 5.98±0.60, *P*=0.80) (**b**).

The participants born preterm had significantly increased pulse wave velocity measured by SphygmoCor, Vicorder and cardiovascular magnetic resonance (5.82±0.80 *vs.* 5.47±0.59 m s^−1^, *P*<0.01, 9.06±1.25 *vs.* 8.33±1.28 m s^−1^, *P*=0.01, and 5.23±1.19 *vs.* 4.75±0.91 m s^−1^, *P*<0.01, respectively, Figure 2a). Those born preterm also had significantly reduced carotid distensibility (4.75±1.31 *vs.* 5.60±1.48 mm Hg^−1^10^3^, respectively, *P*<0.001), but not brachial distensibility (1.07±0.48 *vs.* 1.19±0.54 mm Hg^−1^10^3^, respectively, *P*=0.12), and had a significantly higher augmentation index (6.85±9.64% *vs.* −4.01±11.83%, respectively, *P*<0.001). However, in a multivariate analysis of measurements of arterial stiffness, the magnitude and significance of the differences in pulse wave velocity and distensibility were markedly attenuated after the inclusion of mean blood pressure (Table 2). To verify these findings, we used CAVI, which provides an index of global arterial stiffness independent of blood pressure at the time of measurement. In the subgroup with CAVI measurements (*n*=27 preterm and *n*=94 term), there were no significant differences in arterial stiffness between the groups (5.95±0.72 *vs.* 5.98±0.60 CAVI units, respectively, *P*=0.80, Figure 2c).

There was a moderate inverse relationship between the augmentation index and both the thoracic and abdominal aortic luminal area (*r*=−0.39 *P*<0.001 and −0.54 *P*<0.001, respectively). There was a significant correlation between the central mean arterial pressure and abdominal aortic size as well as a trend for an association between amplification from the central to brachial systolic blood pressure and abdominal aortic area (*r*=0.20, *P*=0.05) but not the thoracic aortic area (*r*=-0.02, *P*=0.85) in those born preterm. However, the coefficient suggests only ~4% of the variation in blood pressure is related to aortic size, with a trend for an even smaller degree related to variations in thoracic aortic diameter (*r*=-0.19, *P*<0.01 and −0.10, *P*=0.08, respectively). There was no consistent relationship between measurements of pulse wave velocity or peripheral distensibility and aortic luminal area. Therefore, any association is likely to be driven by factors common to both aortic size and changes in wave reflection independent of arterial stiffness per se. There is a relationship between gestational age and thoracic (but not abdominal) aortic size in the preterm cohort (*r*=0.25, *P*=0.02) that is consistent with an impact of preterm birth on aortic development but not with aortic stiffness (pulse wave velocity measured using SphygmoCor *r*=−0.02, *P*=0.86; Vicorder *r*=−0.08, *P*=0.70; cardiovascular magnetic resonance (CMR) *r*<0.01, *P*=0.98).

## Discussion

The aortic lumen cross-sectional area is markedly reduced in adults born preterm. Importantly, this reduction in aortic size is out of proportion to the body surface area, which is similar in the two groups. In contrast, the carotid and brachial arteries were not smaller, and despite the localized changes in aortic size, the increased pulse wave velocity in those born preterm could largely be accounted for by their higher mean blood pressure.

The differences in aortic size are in keeping with previous reports of reduced vessel size in children and adolescents born preterm.^4, 14, 33^ A disproportionate reduction in the size of other areas of the cardiovascular system has also been observed.^6, 7^ These findings suggest that preterm birth could lead to a common modification of the cardiovascular developmental pathway.^34^ However, our findings suggest that conduit vessels may respond differently to preterm birth compared with the aorta. We measured multiple locations within the vasculature within each participant and found that differences in aortic size did not reliably indicate ubiquitous vascular changes. Central vessels are substantially richer in elastin content. We previously observed that exposure to antenatal glucocorticoids, which were known to have a potent effect on connective tissue maturation, resulted in a very localized reduction in aortic arch function.^9^ The preterm aorta may also be preferentially susceptible due to the marked local vascular hemodynamic changes at birth, such as the cessation of umbilical flow and increase in left ventricular output.^6^ Additionally, the use of cannulae through the umbilicus in some preterm neonates could influence aortic development during the first weeks of life. Those born preterm have significantly higher blood pressure than those born at term. However, only a small proportion of this population is associated with differences in aortic geometry because there was only a weak relationship between abdominal or thoracic aortic size and central blood pressure.

Despite significant localized changes in aortic size, there appears to be only small differences in aortic stiffness. Although pulse wave velocity differed significantly between the groups, the degree of change was relatively small (0.3–0.7 m s^−1^). In a previous study, we reported a similar degree of variation in pulse wave velocity based on a single tonometry-derived index in adults born preterm whose mothers were normotensive.^22^ In the current study, we used three different pulse wave velocity technologies, each with unique approaches for the assessment of arterial stiffness (from cardiovascular magnetic resonance flow profiles to tonometry-derived carotid-femoral measures and sphygmomanometer-derived brachial-femoral pulse wave velocity). All three showed similar degrees of change. We previously reported that perinatal management, such as the use of antenatal steroids and intravenous lipid feeding regimens, could have an impact on different regions of the aorta.^9, 10^ Taken together, these findings suggest arterial stiffness does differ in early adulthood in association with a preterm perinatal history.^22^ However, data from the ENIGMA study of young healthy adults demonstrated that carotid-femoral pulse wave velocity increased by 0.5 m s^−1^ for each 10 mm Hg increase in mean arterial pressure,^35^ and reference values published by the Arterial Stiffness Collaboration in 2010^36^ described a beta coefficient of mean arterial pressure with a pulse velocity of 0.047 m s^−1^ for a 1 mm Hg rise in a population with a similar age to our study population. Therefore, the majority of the differences in pulse wave velocity (PWV) demonstrated in this study could be accounted for by the variation in blood pressure between groups rather than true pathological differences in arterial stiffness; this possibility was confirmed in our multivariate analysis. This result would be consistent with the findings of the EPICURE follow-up study of school age preterm children^17^ in which there was a relatively small difference in blood pressure and no evident difference in aortic stiffness.

Furthermore, we found no difference in the CAVI between the groups. CAVI is a technique that is considered to be independent of blood pressure at the time of measurement.^30, 31^ It is possible that the CAVI was unchanged in our study because it measures ‘global' stiffness and incorporates both changes in central aortic stiffness and smaller, more muscular arteries. As a result, those born preterm could have a compensatory increase in the distensibility of muscular arteries in response to increased central aortic stiffness, as has been observed in hypertensives.^37^ However, we found no difference in brachial distensibility and a slight reduction in carotid distensibility. The arterial measurement with the greatest proportional difference was the augmentation index; we also observed a trend for lower amplification of systolic blood pressure in those born preterm. The augmentation index was closely related to the reduced aortic size; although aortic size did not account for differences in blood pressure, we recently reported that reduced microvascular structure was related to both preterm birth and blood pressure.^32^ The augmentation index is determined by both central arterial stiffness and changes in the peripheral vasculature, which can have a significant impact on the augmentation index in the absence of changes in central arterial stiffness.^38^ Therefore, more widespread changes in vascular structures related to preterm birth might partially explain the differences in the augmentation index and blood pressure.^39^

To the best of our knowledge, this is the largest cardiovascular magnetic resonance study of preterm offspring. Cardiovascular magnetic resonance is an accurate and safe modality for measuring aortic dimensions, although it should be noted that it is not as precise as some other non-invasive techniques in terms of temporal resolution for the assessment of arterial stiffness.^40^ However, by combining CMR with other non-invasive measurements we were able to undertake a comprehensive assessment of multiple functional and structural macrovascular measurements in adults. The detailed information available has allowed us to take into account the effects of body size (41) and blood pressure^3, 4, 5^ on vascular measurements. A potential weakness of the study was that several technologies became available during the course of our follow-up. Therefore, some of the arterial stiffness measures (such as CAVI) were available in only a subgroup. However, the findings from these measurements are in accord with those performed in the full cohort. Because we do not have measurements of vessel size in childhood, it is possible that all vessels were originally smaller but that the conduit vessels have undergone a compensatory adaptation in adulthood. Longitudinal studies of the vasculature may be of value to study this hypothesis.

In conclusion, there is a localized reduction in aortic size in adults born preterm, although the carotid and brachial arteries are not smaller. Nevertheless, this finding does not result in clinically relevant changes in global arterial stiffness, as assessed by pulse wave velocity, and it seems unlikely that changes in arterial stiffness account for increased cardiovascular and cerebrovascular risk in adults born preterm. However, these risks may increase later in life. Those born preterm have elevated blood pressure from a young age and therefore may be particularly prone to accelerated vascular ageing.

## Figures and Tables

**Table 1:** Characteristics of Cohorts

	*Preterm-born young adults (*n*=102)*	*Term-born young adults (*n*=102)*	P*-value*	*CAVI subgroup (*n*=27)*
*Demographics*				
Age, y	25.1±1.4	25.0±2.6	0.763	26.6±1.1
Gestational age, wk	30.3±2.5	39.6±0.9		30.4±2.8
Males, *n* (%)	47 (46.1)	47 (46.1)	>0.99	15 (56)
Smokers, *n* (%)	20 (19.6)	20 (19.6)	>0.99	3 (11)
				
*Anthropometrics*				
Birth weight, g	1297.0±286.8	3460.0±417.0		1310.0±306.2
Height, cm	169.1±10.0	173.5±9.0	<0.001	169.7±10.5
Weight, kg	73.0±20.5	69.3±12.5	0.33	73.4±16.9
BMI, kg m^−2^	24.9±5.4	22.9±3.1	0.003	25.5±5.7
BSA, m^2^	1.81±0.21	1.83±0.20	>0.99	1.84±0.22
Waist/hip ratio	0.78±0.10	0.81±0.06	0.91	0.84±0.07
				
*Brachial Blood Pressure (mm Hg)*				
Systolic	121.3±10.9	112.9±10.1	<0.001	125.5±9.6
Diastolic	73.0±7.2	68.8±7.0	<0.001	75.8±8.3
Mean arterial pressure	89.1±7.4	83.5±7.1	<0.001	92.3±7.7
Pulse pressure	48.4±9.1	44.1±8.5	0.001	49.7±8.8
				
*Central blood pressure (mm Hg)*				
Systolic	108.0±10.0	97.5±8.8	<0.001	109.8±9.3
Diastolic	74.5±7.4	69.6±7.7	<0.001	76.7±8.2
Mean arterial pressure	85.7±7.7	78.9±7.6	<0.001	87.7±8.0
Pulse pressure	33.5±7.1	27.9±5.6	<0.001	33.1±6.5

Abbreviations: BMI, body mass index; BSA, body surface area; CAVI, cardio-ankle vascular index.

Values as are given as mean±s.d. unless stated otherwise. *P*-values relate to differences between the full cohort of preterm and term-born adults adjusted for age and sex.

**Table 2:** Results

	*Preterm-born young adults (*n*=102)*	*Term-born young adults (*n*=102)*	P*-value*	P*-value in multivariate analysis*^a^
*Aorta structure*				
Thoracic aorta (cm^2^)	2.19±0.44	2.69±0.60	<0.001	<0.001
Abdominal aorta (cm^2^)	1.25±0.36	1.94±0.45	<0.001	<0.001
				
*Conduit structure*				
Carotid artery (mm^2^)	23.55±3.84	23.87±3.55	0.64	0.76
Brachial artery (mm^2^)	9.34±3.25	8.57±2.91	0.10	0.001
				
*Arterial stiffness*				
SphygmAcor PWV	5.82±0.80	5.47±0.59	<0.01	0.07
Vicorder PWV	9.06±1.25	8.33±1.28	0.01	0.05
CMR PWV	5.23±1.19	4.75±0.91	<0.01	0.04
CAVI	5.95±0.72	5.98±0.60	0.80	0.65
Carotid distensibility (mmHg^−1^10^3^)	4.75±1.31	5.60±1.48	<0.001	0.08
Brachial distensibility (mmHg^−1^10^3^)	1.07±0.48	1.19±0.54	0.12	0.77
Augmentation index	6.85±9.64	−4.01±11.83	<0.001	<0.001

Abbreviation: CAVI, cardio-ankle vascular index.

Values as are given as mean±s.d. unless stated otherwise. *P*-values relate to differences between the full cohort of preterm and term-born adults adjusted for age and sex.

a For aortic and conduit vessel measures *P*-value reflects a multivariate model including height and mean blood pressure and for measures of stiffness *P*-value reflects the difference in a model including mean blood pressure.

## Notes

Dr H Boardman has received an unrestricted grant from Fukuda Denshi (Tokyo, Japan) who manufacture and market VaSera.
