# Correction: Corrigendum: Comprehensive multi-modality assessment of regional and global arterial structure and function in adults born preterm

**DOI:** 10.1038/hr.2016.36

**Published:** 2016-04-14

**Authors:** Henry Boardman, Katherine Birse, Esther F Davis, Polly Whitworth, Veena Aggarwal, Adam J Lewandowski, Paul Leeson

**Keywords:** Hypertension, Preterm birth

**Correction to:** *Hypertension Research* (2016) **39**, 39–45; doi:10.1038/hr.2015.102

This article was originally published under a CC BY-NC-ND v4.0 license, but has now been made available under a CC BY 4.0 license. The PDF and HTML versions of the paper have been modified accordingly.

